# Sediment Characteristics in Stonewort *Chara tomentosa* Assemblages

**DOI:** 10.1002/ece3.72772

**Published:** 2026-01-17

**Authors:** Irma Puttonen, Henna Raitanen, Miriam Nystrand, Sonja Salovius‐Laurén

**Affiliations:** ^1^ Environmental and Marine Biology Åbo Akademi University Turku Finland; ^2^ Geology and Mineralogy Åbo Akademi University Turku Finland

**Keywords:** Åland, Baltic Sea, Charophyta, geochemistry, priority habitat, restoration, threatened species

## Abstract

Charophytes are green macroalgae that lay the foundation of important habitats in shallow, low‐salinity, and sheltered areas of the Baltic Sea. Charophyte habitats provide food, shelter, and reproduction sites for diverse benthic communities, fish, and waterbirds. Due to human pressures, charophytes have declined in the Baltic Sea. They are sensitive to water turbidity and eutrophication. To protect and restore charophytes and their habitats, knowledge of the species' environmental requirements is fundamental. We examined differences in sediment and porewater properties in areas with and without the large charophyte species *Chara tomentosa* in five shallow sheltered bays in the northern Baltic Sea. Sediment samples were analysed for total contents of phosphorus (P), nitrogen (N) and carbon (C), grain size, water content, loss‐on‐ignition, and a set of easily soluble elements (Al, B, Ba, Ca, Cd Co, Cr, Cu, Fe, K, Mg, Mn, Na, Ni, P, Pb, S, Sr., and Zn) from deionised water extraction. From the porewater, dissolved inorganic P (DIP) and N in nitrate and nitrite (NO_2,3_‐N) were analysed. The results showed significantly lower porewater DIP concentrations and finer grain sizes in the sediments from areas with *C. tomentosa* compared to those without it. The variability of the organic content and the total contents of P, N, and C was lower in the sediments from the seafloor areas occupied by *C. tomentosa* than in those without it. The contents of easily soluble elements were similar in all sediments. We conclude that the sediment grain size and organic content should be considered in charophyte restoration. The results show that *C. tomentosa* habitats influence phosphorus cycling in the sediment–water interface. How microbial communities associated with charophytes influence phosphorus cycling needs further attention to estimate the link between charophyte habitats and eutrophication.

## Graphical abstract

The study investigated sediment characteristics associated with Chara tomentosa. The results show that sediment grain size and the concentration of the dissolved phosphate in the porewater differ in the seafloor areas characterised by *C. tomentosa* from adjacent areas at similar depth, but without charophytes. The invaluable charophyte habitats are threatened in the shallow coastal bays and lagoons of the Baltic Sea. A thorough understanding of the species' environmental requirements, including the sediments, is fundamental for successful restoration.

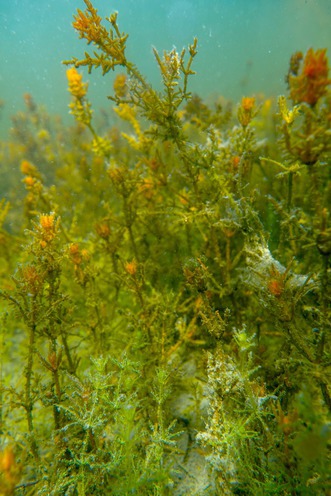

## 1 Introduction

The seafloor substrate forms the foundation for marine benthic habitats. The origin of coastal sediments is a heterogeneous mixture of riverine discharge from the catchment, sediments transported by waves and currents, and autochthonous organic and minerogenic material. Bedrock and soils in the catchment, influenced by land use, form a major part of the sediments that rivers transport to the sea. Multiple and complex processes in the coastal zone reform the sediments' physical and chemical properties (McCave 1984).

Soft sediments in shallow shelf areas accommodate submerged macrophytes that can attach to the substrate and acquire nutrients from it. Coastal sediments, therefore, play an important role in macrophyte species distribution (Selig et al. 2007). The sediment characteristics, such as grain size, density and cohesive strength, water content, organic matter content, and nutrient contents and availability, frame and modify the structure and composition of the underwater macrophyte communities (Bornette and Puijalon 2011; Selig et al. 2007; Silveira and Thomaz 2015). Conversely, macrophytes interact with their substrate through growth, metabolism, and decomposition (Madsen et al. 2001). Sediment nutrient contents modify macrophytes' morphology by adjusting biomass allocation to their roots or shoots, depending on sediment nutrient availability (Wang et al. 2008; Xie et al. 2013). These changes impact macrophytes directly and indirectly, e.g., through competition. High nutrient content in sediment and porewater may deteriorate macrophyte community health (Carling et al. 2013), thus weakening their survival. Depending on the species and its requirements, sediment type can aid or hamper macrophyte re‐establishment and reproduction (Jiang et al. 2008). Moreover, macrophyte communities composed of different macrophyte species host habitat‐specific microbial, plankton, zoobenthos and fish communities, and they all have their special role in the functioning of a diverse and resilient ecosystem (Hempel et al. 2008; Kataržytė et al. 2017). Environmental conditions, such as light availability, temperature, salinity, and pH in the water, have been shown to shape and structure macrophyte communities (Bornette and Puijalon 2011); however, the role of substrate composition and properties remains poorly understood.

Charophytes (Charales) are key macroalgal species in the submerged macrophyte communities in the brackish‐water Baltic Sea sheltered coastal bays and lagoons. Their complex morphology resembles that of vascular plants, and they are considered closely related to photosynthesising land plants. Most charophytes are preferentially freshwater species, but some species, such as *Chara aspera*, *C. baltica* , *C. canescens* and *C. tomentosa* , occur in brackish water, and some (i.e., *Lamprothamnium* spp.) even in hypersaline environments (Garcia and Chivas 2006; Pukacz et al. 2024). Each charophyte species has a specific salinity tolerance (Rey‐Boissezon and Auderset Joye 2015). In the Baltic Sea, low salinity gives rise to diverse submerged macrophyte communities of marine and freshwater species while causing stress to both, as the species live at the limit of their salinity tolerance.

Charophyte habitats are invaluable, as they provide food, protection from predation and sheltered reproduction sites for diverse communities of periphyton, plankton, macroinvertebrates, insects, fish, and waterbird species (Eveleens Maarse et al. 2024; Snickars et al. 2010). Charophytes bind carbon and nutrients in photosynthesis, and as they attach to their substrate, they stabilise the sediment, thus reducing resuspension and water turbidity. In freshwater and low‐salinity environments, charophytes accumulate a carbonate encrustation on their thallus, slowing down the decomposition of the biomass; hence, retaining the nutrients accumulated in their biomass in the sediments for a long time, and thereby acting as nutrient sinks. Moreover, although the carbonate encrustation declines in rising salinity (Herbst et al. 2018), it may accumulate in the sediments (Pełechaty et al. 2013) and co‐precipitate phosphorus into the sediments permanently (Siong and Asaeda 2006).

Due to human activities, charophyte habitats have declined in the Baltic Sea during the past decades (Pitkänen et al. 2013). Eutrophication caused by extensive nutrient runoff from, e.g., agriculture and wastewater, is still a major threat to the coastal biodiversity of the Baltic Sea (Sumelius et al. 2025; Voss et al. 2011), and charophytes are particularly sensitive to eutrophication (Blindow 1992). High nutrient concentration in the water enhances primary production in the sea, leading to extensive and harmful algal blooms and oxygen depletion. Eutrophication has altered the benthic habitats in many ways in the Baltic Sea (Bonsdorff et al. 1997), and the changes are likely to be reflected in the sediments. Elevated contents of certain elements, such as copper (Cu), cadmium (Cd), cobalt (Co), nickel (Ni), boron (B), and manganese (Mn) in sediments can be toxic and inhibit charophyte growth (Lambert and Davy 2011). Heavy metal contamination from industrial sources is a widespread issue in the Baltic Sea sediments (Manzetti 2020). Moreover, acid sulphate soils can release significant amounts of heavy metals into the coastal area of the Baltic Sea, impacting the water and sediment quality and the aquatic life (Hudd and Kjellman 2002; Nordmyr et al. 2008; Nystrand et al. 2016). Coastal development and activities like turbulence caused by boat traffic, construction, or dredging further damage sensitive charophyte meadows (Sagerman et al. 2020; Schubert and Blindow 2004).

**FIGURE 1:**
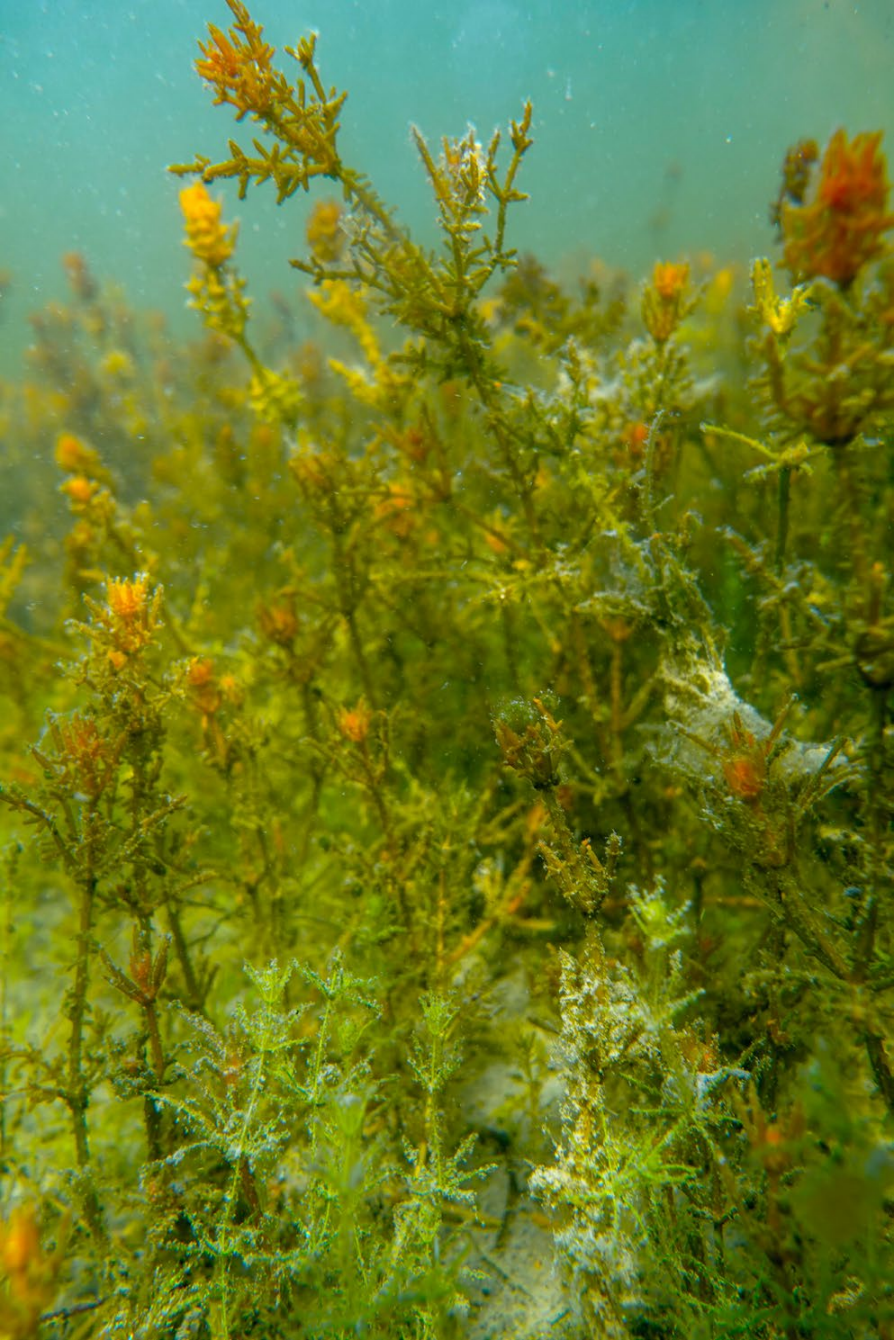
Large, uniform charophyte meadows have declined in the shallow coastal bays and lagoons of the Baltic Sea. Charophyte habitats are listed as a priority habitat in the European Union Habitats Directive. Photo: Roxana Preston.

Coastal lagoons, often containing red‐listed charophyte species (Figure 1) (Schubert and Telesh 2017), are among the priority habitat types in the European Union (EU) Habitats Directive (Directive 92/43/EEC 1992) due to their ecological significance. Therefore, restoration of these invaluable habitats has been piloted, but with varying or little success (Faithfull et al. 2024).

Marine habitat conservation and restoration goals are set at global, EU and national levels. In 2022, 190 countries adopted the Kunming‐Montreal Global Biodiversity Framework (GBF). One of its ambitious targets is to restore 30% of all degraded marine ecosystems by 2030, and by 2050, ensure that the integrity, connectivity and resilience of all ecosystems are maintained, enhanced, or restored. The EU Biodiversity Strategy for 2030 and the EU Nature Restoration Law (EU 2024/1991) form the framework for EU countries to implement the GBF. To achieve these goals, there are attempts both to restore and conserve these habitats. Sheltered benthic habitats characterised by Charales are protected by the Nature Conservation Act in Finland, and red‐listed by The Convention on the Protection of the Marine Environment of the Baltic Sea Area (HELCOM 2013).

Knowledge of the environmental requirements is a prerequisite for a successful restoration of declining species. Water quality sets limits to charophyte survival (Rodrigo and Alonso‐Guillén 2013), as the water turbidity, turbulence, and sediment accumulation weaken them (Henricson et al. 2006). Many studies on aquatic plants show that the sediment quality influences macrophyte growth (e.g., Carling et al. 2013; Handley and Davy 2002; Jiang et al. 2008; Rodrigo et al. 2013). Often, the sediment quality has been determined only by visual inspection (Kautsky et al. 1999; Torn et al. 2004, 2019). A more detailed characterisation of the sediments associated with charophytes is therefore needed. We investigated the sediment quality related to *Chara tomentosa*, one of the largest charophyte species in coastal lagoons and shallow, sheltered bays in the northern Baltic Sea.

We compared the sediments in charophyte meadows with those in areas without charophytes within the same bays. The hypothesis is that there is a difference in the sediment's physical and/or chemical properties between the sites dominated by *C. tomentosa* and those occupied by other macrophytes but with no charophytes.

## 2 Materials and Methods

### 2.1 Study Area

**FIGURE 2:**
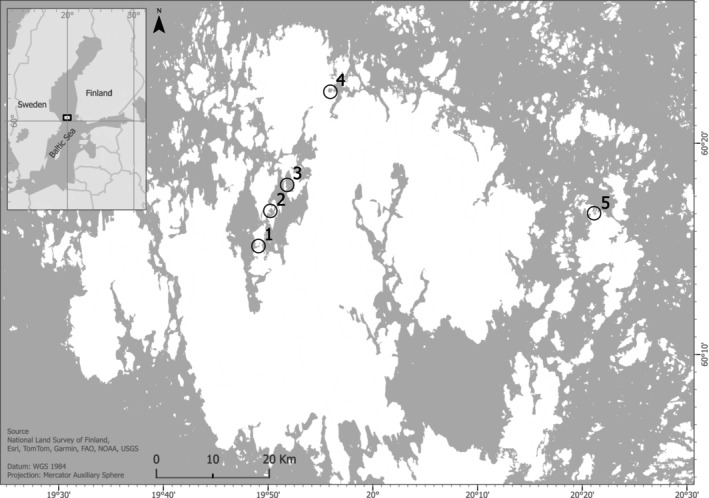
Sediment and water samples were collected from five shallow, sheltered bays in the Åland inner archipelago.

Sediment and water quality were studied in five sheltered, shallow bays where the charophyte species *C. tomentosa* has been growing for at least the past 20 years (Ikonen 2023). The study area is in the Åland Islands archipelago between the Baltic Proper and the Bothnian Sea (Figure 2).

The sediments transported from the drainage area into the bays originate from the soils. The mineral component of the soils is composed of weathered bedrock and till. Åland Islands' bedrock consists mainly of Proterozoic orogenic rapakivi granite (Winterhalter et al. 1981). Coniferous and mixed forests and agricultural land dominate land use in the catchment of the studied bays, labelling the organic material and nutrient profile in the sediments. The studied bays are sheltered; slow water exchange between adjacent sea areas is characteristic of them, and the bay waters are influenced by inflow from the relatively small catchments. In all bays, common reed, *Phragmites australis* , dominates the shoreline.

### 2.2 Sediment, Porewater and Surface Water Sampling

Sediment, porewater and surface water samples were collected at two sampling stations in each bay (Figure 2). One of the sampling stations was dominated by *C. tomentosa* and the other was dominated by various macrophytes, but no charophytes. Hereafter, the ten sampling stations from the five bays are referred to as with and without charophytes, respectively. Two sediment samples and one porewater sample were collected at each sampling station. The sampling was done to examine potential differences between the sediments from areas with and without charophytes. To minimise potential effects of water depth and exposure to waves and currents, the sampling stations in each bay were selected as close to each other as possible. The water depth at the sampling stations ranged between 60 and 198 cm (Table 1). In Bay 4, we did not find an area with and without charophytes at similar depths; hence the greater depth difference between the sampling stations in Bay 4 (Table 1). Macroscopic plant and algae species growing on the seafloor were surveyed and logged by snorkelling.

**TABLE 1:** The water depth at the sampling stations and the measured water quality variables in the studied bays at the time of the sampling.

Bay number	1	2	3	4	5
Surface area (km^2^)	0.04	0.12	0.93	0.13	0.04
Coordinates (WGS84)					
With charophytes	60.2524°N 19.8180°E	60.2800°N 19.8368°E	60.3005°N 19.8636°E	60.3739°N 19.9324°E	60.2782°N 20.3521°E
Without charophytes	60.2521°N 19.8183°E	60.2802°N 19.8373°E	60.3015°N 19.8636°E	60.3748°N 19.9302°E	60.2790°N 20.3521°E
Water depth—with charophytes (m)	0.64	1.10	1.15	1.05	0.75
Water depth—without charophytes (m)	0.60	1.22	1.15	1.98	0.75
Water temperature (°C)	14.3	18.7	16.3	16.2	15.4
DO (mg L^−1^)	5.41	9.96	8.13	10.17	7.55
Conductivity (mS cm^−1^)	8.98	8.77	8.53	9.08	9.84
Salinity	5.02	4.92	4.77	5.11	5.57
pH	6.97	7.93	7.57	8.15	6.93
Chl‐a (μg L^−1^)	6.27	15.49	8.65	4.33	19.69
Turbidity (NTU)	1.84	3.77	1.88	1.22	2.04
TN (μg L^−1^)	812.0	820.1	745.3	540.5	606.2
NO_2,3_‐N (μg L^−1^)	2.5	1.8	10.5	59.6	1.6
TP (μg L^−1^)	33.0	48.9	33.3	28.9	46.1
DIP (μg L^−1^)	11.6	12.7	11.7	7.6	15.2

Abbreviations: Chl‐a, chlorophyll‐a; DIP, dissolved inorganic phosphorus; DO, dissolved oxygen; NO_2,3_‐N, dissolved nitrogen in nitrite and nitrate; TN, total nitrogen; TP, total phosphorus.

Sediment samples were collected with a polypropene sewage pipe with an inner diameter of 70 mm. The pipe was pushed through the macrophytes, at some places dense and thick, to the sediment, closed with a tight cap and lifted to the boat. The cap was removed, and the free water on top of the sediment was poured slowly out. The uppermost 10 cm of the sediment sample was then recovered into an airtight plastic bag and sealed after air removal. Two sediment samples were recovered at each station and stored in a freezer at −19°C until the analyses. A third sediment sample was collected for porewater analysis. One porewater sample was collected at each study station by filtering with a Rhizon CSS sampler, distributed by Rizosphere Research Products, Netherlands, from a 5 cm depth in the sediment. The volume of the porewater samples was 5–10 mL.

Surface water samples from the water column were collected above the macrophytes with a Limnos water sampler to explore the water quality in the bays. One surface water sample was collected from Bays 1, 2, and 4 between the sediment sampling stations, and from Bays 3 and 5, two surface water samples were collected at approximately 100 m distance from each other. The water samples were stored in LD polyethene bottles. Surface water turbidity was analysed on the sampling day. All other samples were frozen and stored at −19°C until the analyses.

The YSI EXO3 Multiparameter Water Quality Sonde was used to measure the temperature (T), pH, dissolved oxygen (DO), conductivity, and salinity during the sampling above the macrophyte communities at each sampling station.

All samples were collected on 12–30 September 2023, and the air temperature at the sampling time ranged between 14°C and 19°C.

### 2.3 Sediment Analyses

The physical characteristics of the sediments were studied by determining the water content, organic matter content, and grain size distribution. The water content of the sediments was determined from approximately 5 g sub‐samples by oven‐drying at 105°C for 24 h. The organic matter content was estimated as loss‐on‐ignition (LOI) by combusting a dried sample in a muffle furnace at 550°C for 4 h (Radojević and Bashkin 1999). Grain‐size distribution was analysed using the hydrometer method (Ashworth et al. 2001). Before the analysis with the hydrometer method, the samples were bathed in 30% hydrogen peroxide and heated to 65°C until the frothing ceased, to remove organic material. The samples were then washed by adding 150 mL deionised water and 15 mL 2 M hydrochloric acid (HCl) and stirring for 1 min. After washing, the samples were left to settle overnight and decanted the following day. A dispersion agent was used to avoid flocculation of the particles. 100 mL 0.05 M sodium pyrophosphate solution and 200 mL deionised water were added, and the samples were shaken for 2 h, then left to settle overnight and shaken again for 2 h. Grain‐size distribution is expressed as weight percentages of sand (Ø 0.063–2 mm), silt (0.002–0.063 mm) and clay (< 0.002 mm).

Contents of 19 elements (Al, B, Ba, Ca, Cd Co, Cr, Cu, Fe, K, Mg, Mn, Na, Ni, P, Pb, S, Sr., and Zn) were analysed from Milli‐Q water extracts of the sediment samples using the Inductively Coupled Plasma‐Optical Emission Spectroscopy (ICP‐OES) method (Khan et al. 2022) at the laboratory for Analytical Chemistry at Åbo Akademi University (Turku, Finland). The samples for ICP‐OES analysis were conserved with 2–3 drops of 65% ultrapure nitric acid, HNO_3_. The ion chromatography (IC) method was used to determine the contents of sulphate (SO_4_^2−^), chloride (Cl^−^), phosphate (PO_4_^3−^) and nitrate (NO_3_^−^) ions in the sediments. Two replicate samples were prepared for ICP‐OES and IC analyses. The analytical precision (Gill 2014), based on 7 duplicates, was within 10%.

Instead of acid digestion, we used extraction with Milli‐Q water to determine the easily soluble element contents, which were considered relevant for charophyte growth. The leachate method used resembles the methods presented by Mattbäck et al. (2022) as it was modified from the EN 12457–2 one‐step 10:1 liquid–solid leaching method. In the modified method, approximately 8 g of each sediment sample were weighed into a sterile 50 mL centrifuge tube. 30 mL of Milli‐Q water was added, and the tubes were placed on a Vortex shaker for 1 h for the one‐step 10:1 liquid–solid extraction. The samples were then centrifuged for 15 min at 4300 rpm. The extracted solution was filtered through a 0.2 μm filter into 15 mL sterile centrifuge tubes, one for ICP‐OES and one for IC analysis. Five grams of each wet sample were weighed in a crucible and dried at 105°C overnight to determine the sample's water content. The dilution of each sample was calculated from the dry weight (dw) of the sample. The results are presented as mg kg^−1^ dw.

Freeze‐dried sub‐samples were sent to the Tvärminne Zoological Station laboratory for the analysis of the sediments' total contents of phosphorus (TP) with a spectrophotometric method (Koistinen et al. 2020a) and carbon (TC) and nitrogen (TN) with a mass spectrometer (Koistinen et al. 2020b, 2020c).

### 2.4 Surface Water and Porewater Analyses

Surface water quality was analysed to define the water quality status in the studied bays. Turbidity was analysed immediately after sample collection with a Hach 2100P Turbidimeter. Chlorophyll‐a (Chl‐a) samples were filtered immediately after sample collection, and the filters were stored in a freezer. The Chl‐a concentration was analysed spectrophotometrically with a ThermoSpectronic Aquamate Spectrophotometer using the ethanol extraction method within 2 months.

At Husö, TP, TN, and dissolved inorganic phosphorus (DIP) and nitrogen in nitrite and nitrate (NO_2,3_‐N) were analysed from the surface water and porewater samples with a spectrophotometer following the APHA AWWA WEF standards. The surface water samples were filtered through a 0.22 μm cellulose acetate filter before the DIP and NO_2,3_‐N analyses. The porewater samples were filtered during sampling through a 0.15 μm filter in the membrane of the Rhizon sampler.

### 2.5 Statistical Analyses

To test the hypothesis that the mean values in the analysed sediment and porewater variables (sediment leached elements and ions, TP, TN, TC, grain size, organic content, and porewater PO_4_‐P and NO_2,3_‐N) differed between the two groups, i.e., sediments from areas with and without charophytes, a permutation test (Unseld et al. 2025) was applied. A Perm_diffs vector with 10,000 permutations was calculated in R software, v.4.2.2. The non‐parametric permutation test was selected because the data did not meet the assumptions of normal distribution and homogeneity of variances for parametric statistical tests. The results were considered statistically significant when the *p*‐value was less than 0.05.

A Principal Component Analysis (PCA) was used to identify patterns and the most influential variables driving the differences in the data between the groups with and without charophytes. The variables included in the PCA were the leached elements and ions, TP, TN, TC, the content of fine particles (silt and clay) and LOI in the sediment. The samples were divided into two groups (with and without charophytes).

## 3 Results

### 3.1 Macrophyte Species and Surface Water Quality

Charophytes were found in all five studied bays. *C. tomentosa* was the dominant charophyte species at our sampling stations, and occasional *C. baltica* individuals were observed at the sampling stations in Bays 2 and 4. However, at the sampling station in Bay 5, only sporadic *C. tomentosa* individuals were observed. In Bay 1 at the sampling station without charophytes, an approximately 40 cm thick layer of filamentous algae (*Vaucheria* spp.) covered the seafloor, and other macrophyte species grew through or among the *Vaucheria* layer. The identified macrophyte species in the study area included *Phragmites australis* , *Najas marina* , *Stuckenia pectinata* , *Zannichellia palustris* , *Ceratophyllum demersum* , *Myriophyllum spicatum* , and *Vaucheria* spp.

Although there were some differences in the water quality between the bays, the differences were relatively small between Bays 1, 2, 3, and 5. However, in Bay 4, the DO and NO_2,3_‐N concentrations and pH were higher, and the Chl‐a, TN, TP, and DIP concentrations and turbidity were lower than in any of the other studied bays (Table 1). Moreover, the Chl‐a concentration was higher in Bay 5 than in the rest of the studied bays. As two surface water samples were taken from Bays 3 and 5, the mean values of those are presented in Table 1.

### 3.2 Sediment Properties

**FIGURE 3:**
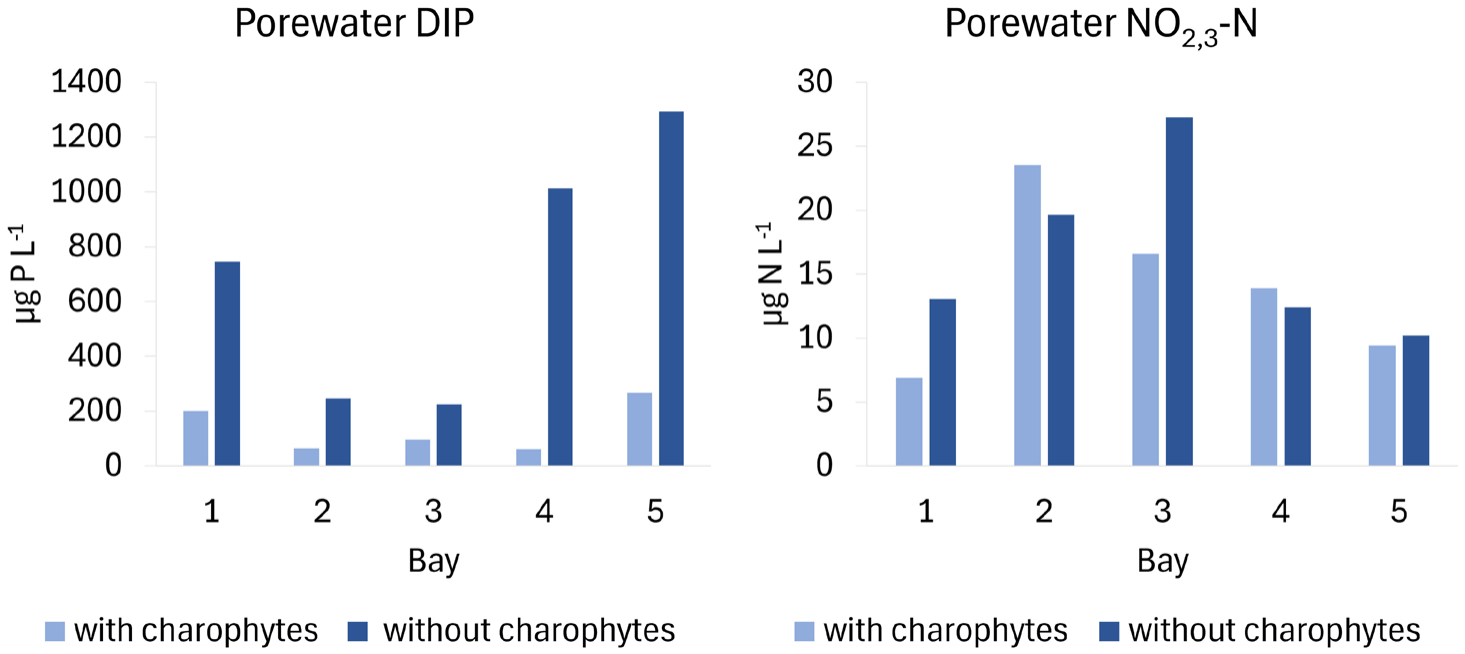
Porewater dissolved inorganic phosphorus (DIP) and nitrogen in nitrate + nitrite (NO_2,3_‐N) concentrations in the sediments from areas with and without charophytes (*n* = 5).

Sediment porewater DIP concentration varied from 17.8 to 266.6 μg L^−1^ within the sampling stations with charophytes. The variation in the sampling stations without charophytes was 225–1293 μg L^−1^ (Figure 3). The mean DIP concentration in the sediment porewater was 566.9 μg L^−1^ lower in the areas with charophytes than those without, and the difference was statistically significant in the permutation test (*p* = 0.023, *n* = 5). The mean dissolved nitrogen concentration in NO_2_ + NO_3_ in the sediment porewater varied between the studied bays (Figure 3), but no statistically significant difference (*p* = 0.604, *n* = 5) was observed between the sediments from areas with and without charophytes.

**FIGURE 4:**
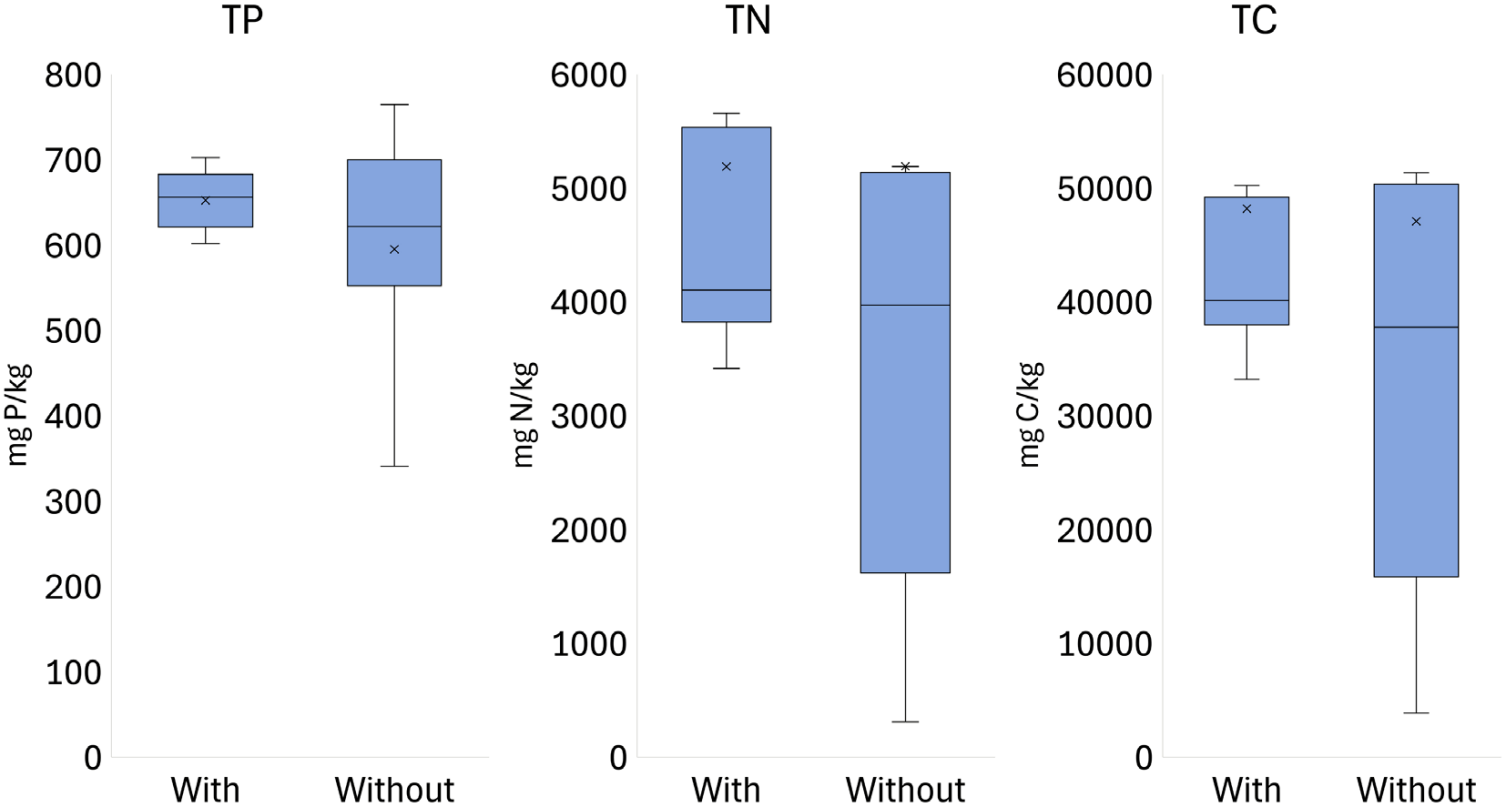
Sediment total phosphorus (TP), nitrogen (TN) and carbon (TC) contents in the areas with and without charophytes (*n* = 10). Boxes: Quartiles, ×: Mean, horizontal line inside the box: Median, whiskers: Minimum and maximum.

The mean contents of total P, N and C in the sediments from areas with and without charophytes were not statistically significantly different (*p* = 0.289, *p* = 0.432 and *p* = 0.367, respectively, *n* = 10; Figure 4). The highest and lowest total contents of P, N, and C in the sediments were observed in the area without charophytes in Bay 4 and 5, respectively. Consequently, the variability of the contents of TP, TN and TC was lower in the sediments from areas with charophytes than those without charophytes (Figure 4).

**FIGURE 5:**
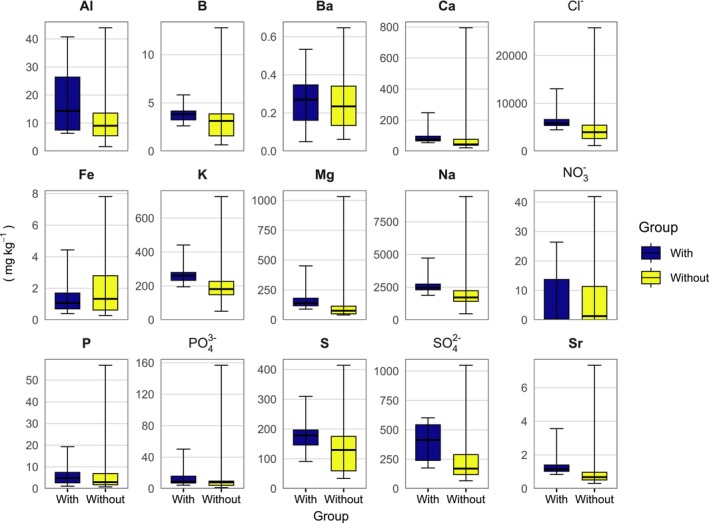
The contents of the leached elements in the sediments by group (with and without charophytes), *n* = 10. Boxes: Quartiles, horizontal line inside the box: Median, whiskers: Minimum and maximum.

There was no statistically significant difference between the areas with and without charophytes in the mean contents of the analysed leached elements. However, in Bay 4, the contents of several leached elements (B, Ca, Cl^−^ K, Mg, Na, P, PO_4_^3−^, NO_3_^−^, S, SO_4_^2−^) were lower in the sediments from areas with charophytes than in those without charophytes. Overall, the variability in these elements was narrower in the sediments from the areas with charophytes (Figure 5). Cd Co, Cr, Cu, Mn, Ni, Pb, and Zn concentrations in the water extract of the sediments were close to or below the detection limit and were omitted (*n* = 10).

**FIGURE 6:**
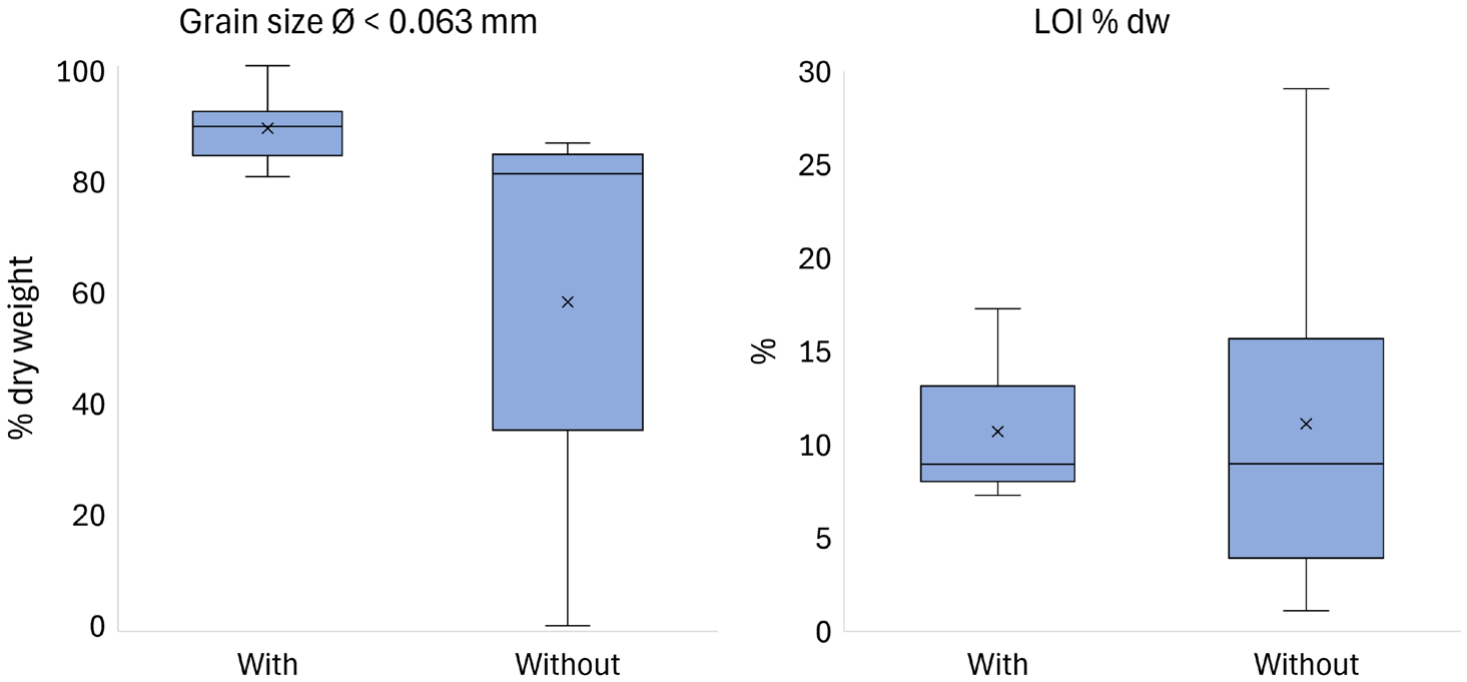
The sediment content of fine particles (particle diameter < 0.063 mm) and loss‐on‐ignition (LOI, % of dry weight) in the areas with and without charophytes (*n* = 10). Boxes: Quartiles, ×: Mean, horizontal line inside the box: Median, whiskers: Minimum and maximum.

The grain size in all sediment samples was below 2 mm. Sediment average content of fine particles (particle diameter < 0.063 mm) was higher in the sampling stations with charophytes than in those without (Figure 6). The difference was statistically significant in the permutation test (*p* < 0.01). The organic content in the sediment, determined as LOI, varied less in the stations with charophytes than in the stations without charophytes. However, there was no statistically significant difference in the mean values between the sediments from areas with and without charophytes (*p* = 0.894) (Figure 6). Moreover, the sediment organic content varied between the studied bays.

**FIGURE 7:**
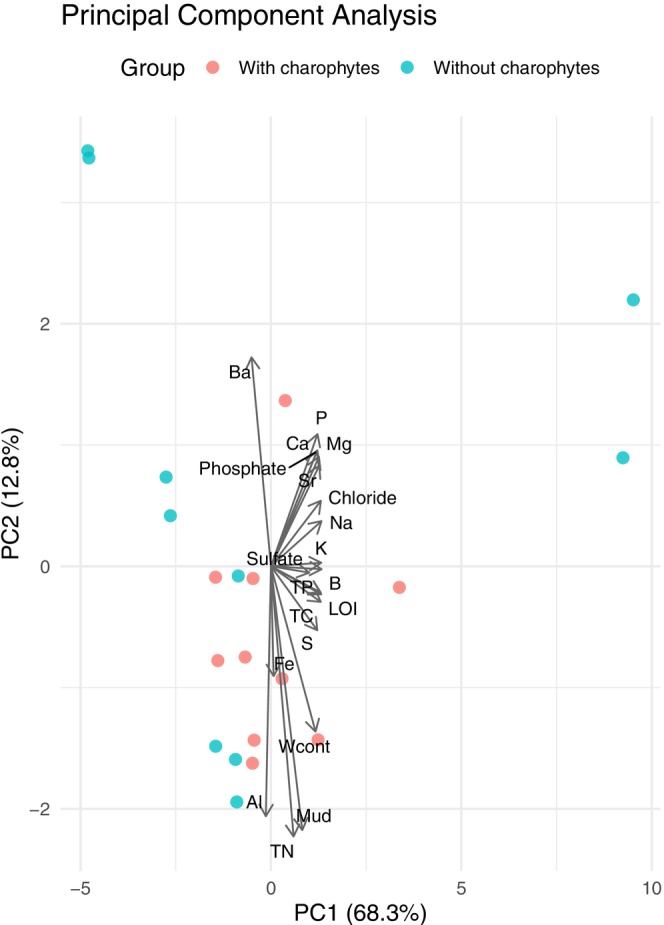
The results of the Principal Component Analysis, including the leached elements and ions in the sediments, Water content (Wcont), Total contents of phosphorus, nitrogen and carbon (TP, TN, and TC, respectively), particle diameter < 0.63 μm percentage (Mud) and organic content as a percentage of sediment dry weight (LOI). The samples are separated into two groups: with and without charophytes. The first Principal Component (PC1) explains 68.3% and PC2 explains 12.8% of the variability in the data.

In the PCA, the first PC explained 68.3% of the variability. The absolute values of the loadings for Fe, Al and Ba were between 0.1 and 0.001, and for the other included variables between 0.12 and 0.27 (Table 2). The second PC explained 12.8% of the variability. Contents of fine particles (Ø < 0.063 mm), TN and leached Al in the sediment correlated negatively with PC2 (loadings −0.44, −0.45, and −0.41, respectively), and the content of leached Ba correlated positively with PC2 (loading 0.34). The samples in the group without charophytes were more widely scattered along the axis than those in the group with charophytes (Figure 7).

**TABLE 2:** The loadings of the input variables in the first two Principal Components (PC) in the Principal Component Analysis.

Variable	PC1	PC2
B	0.266263	−0.00485
Na	0.26576	0.074628
K	0.264608	0.005892
LOI	0.264514	−0.04622
Cl^−^	0.262364	0.108154
TC	0.261825	−0.0587
TP	0.261495	−0.04317
Sr	0.253737	0.166146
Mg	0.252303	0.180684
P	0.244797	0.218017
Ca	0.244	0.191338
S	0.24356	−0.10549
PO_4_^3−^	0.240622	0.189116
Wcont	0.234303	−0.27269
SO_4_^2−^	0.201446	−0.01043
Mud	0.164542	−0.43508
TN	0.118874	−0.4457
Ba	−0.10199	0.344653
Al	−0.02681	−0.41225
Fe	0.014037	−0.18113

## 4 Discussion

Our results revealed differences in the sediment structure and composition in areas where *C. tomentosa* grew compared to sediments where vascular plants or filamentous algae dominated. The porewater DIP concentrations showed the most prominent difference, being lower in the sediments from the areas with charophytes than those without. However, the correlation does not reveal the causality. This result may indicate that *C. tomentosa* changes the porewater phosphate concentration by absorbing nutrients directly from the porewater with their rhizoids or indirectly by taking up phosphate from the water column with their thalli and, consequently, causing a diffusive flux of phosphate from the porewater to the overlying water. Alternatively, *C. tomentosa* may prefer sediment with low DIP concentrations, and other macrophytes outcompete charophytes in high porewater DIP concentrations. In our study area, all sampling stations were covered by macrophytes, either predominantly charophytes or vascular plants with filamentous algae, all taking up nutrients from the porewater or from the water column, or both (Manolaki et al. 2020). Declining DIP concentrations could be explained by the diffusive flux of phosphate from the porewater toward the water column above the sediment surface. During the productive season, the photosynthesising organisms utilise the DIP in the water. During high primary production, when the DIP concentration in the water near the sediment surface is lower than in the porewater in the upper sediment layers, diffusive flux will pump nutrients from the porewater to the water column to reach concentration equilibrium (Lerman 1978). One possible explanation for the lower porewater DIP concentration in the sediments from areas dominated by *C. tomentosa* could be the microbial communities specific to charophytes, which precipitate phosphate minerals within the sediments (Soudry 2000). Alternatively, the microbial communities associated with the aquatic vascular plants and filamentous algae may limit the phosphate flux from the porewater to the water column above the sediment (Soudry 2000). During the time of the sampling, the DIP concentration in the water column was low due to high primary production. In such conditions, it is plausible that nutrient uptake by rhizoids would be more energy efficient than from the low concentration in the water column. A possible explanation could be that high porewater DIP concentration limits the occurrence of *C. tomentosa* , and vascular plants start dominating and outcompete *C. tomentosa* . In freshwater lakes, low phosphorus concentration in the water column can increase *C. tomentosa* prevalence and strengthen its competition over vascular plants (Pełechaty et al. 2015). Our study confirms that DIP concentration in the porewater is linked to *C. tomentosa* distribution, also in brackish water. However, further studies are needed to investigate the factors causing this phenomenon. Experimental studies in controlled conditions would give insight into the interaction of *C. tomentosa* and porewater, and, e.g., if and how the phosphate concentration in the water affects the nutrient uptake in *C. tomentosa* .

Grain size distribution is a basic sediment characteristic (Ashworth et al. 2001). The deposition patterns of fine‐grained sediments are a result of complex processes affected by physical, chemical and biological factors (McCave 1984). Our results indicate that *C. tomentosa* prefers finer sediment grain sizes than other macrophytes at similar water depths. High clay mineral and organic contents are linked to the sediment's high cation exchange capacity (Parfitt et al. 1995; Sidi et al. 2015). Cation exchange capacity is an important factor controlling the sediment's pH buffering capacity and element and nutrient mobility. These properties are important in soil fertility in terrigenous environments (Mengel 1993) and, thus, may influence the distribution of macrophyte communities in coastal environments. Moreover, sediment pH can be an important factor in defining macrophyte communities and needs further attention.

Low mobile heavy‐metal contents indicate that the sediments are not metal‐contaminated in the study area, and that other factors, such as grain size, porewater DIP concentration, light availability and the water chemistry, control the distribution of submerged macrophytes. Sediment resuspension may influence macrophyte community structure by increasing water column turbidity, thus affecting light conditions. Furthermore, elements and nutrients may dissolve by resuspension and the resulting oxidation of the sediment particles.

The narrower range in most analysed element contents in the sediments with charophytes than without suggests that *C. tomentosa* has more restricted criteria for the sediments' geochemistry than the other macrophytes in the area. Although PCA did not reveal a clear indication of a link between the analysed variables and charophytes, it indicated a wider spread of values in the sediments from areas without charophytes than those with charophytes. Studies show that microbes influence the functioning of sediments (Reed and Martiny 2013). A controversial explanation could be that *C. tomentosa* , together with the microbial and infauna communities, reform the mobility of elements. There is evidence of microbe‐mineral interaction in marine sediments (Ransom et al. 1999), and studies show differences in the composition of microbial communities between different macrophyte species (Hempel et al. 2008; Rodrigo et al. 2025). Readily bioavailable DIP in the sediment porewater is regulated by P forms in the sediment, i.e., how P is bonded in chemical compounds, the biodegradability of P forms and bacterial activity transforming P into bioavailable form (Sinkko et al. 2011). Porewater DIP concentration may be influenced by microbial communities that regulate P availability through enzymatic hydrolysis, storage, and redox‐driven transformations (Duhamel 2025; Karl 2014). Several bacterial taxa are known to be involved in P cycling in aqueous sediments, both enhancing P release from and P retention in the sediments. For example, *Pseudomonas*, *Candidatus*, *Luteitalea*, *Gemmatirosa*, and *Granulicella* can increase phosphate solubilisation, whereas *Variovorax*, *Rhodococcus*, and *Luteitalea* may synthesise organic P (Ding et al. 2022). *Candidatus Accumulubacteria* and *Tetrasphaera* can uptake inorganic P (Liu et al. 2018). Charophyte habitats and the associated bacterial communities may be involved in phosphorus cycling, internal loading and eutrophication. In sheltered coastal and archipelago areas, nutrients from riverine discharge accumulate in the vicinity of the river mouths, in sheltered areas, where the water exchange is slow due to the complex topography and abundance of islands (Puttonen et al. 2014). Internal P loading is common, particularly when the degradation of organic material causes hypoxia in the near‐bottom water. In those areas, local controls of eutrophication are crucial for biodiversity and the functioning of habitats.

The concentrations of Chl‐a, TN, and TP in the surface water were all above the threshold values for good ecological status (HELCOM 2023), thus indicating eutrophication in all studied bays. High nutrient concentrations in the water enhance phytoplankton growth, causing turbidity and reduced light penetration, which affect macrophyte growth in different ways (Herkül et al. 2018). Despite eutrophication, charophytes were found in all studied bays, although in Bay 5, only scarce individuals were observed. *C. tomentosa* was the dominant charophyte species in all sites, and some *C. baltica* were also observed. Light availability may restrict charophyte growth in eutrophic systems (Kovtun et al. 2011; Torn et al. 2006). Nevertheless, our results show that in addition to the water quality, sediment characteristics are important for *C. tomentosa* occurrence. Given that eutrophication and decreased light availability due to water turbidity may affect the depth distribution of *C. tomentosa* in our study area, we selected sites with similar depths in each bay to reduce the varying effects of light. In bay 4, however, we could not find optimal areas, and therefore, the depth of the sampling station with charophytes was markedly shallower than that without charophytes. Thus, in Bay 4, the depth may be a more important factor limiting charophyte survival than in the rest of the studied bays.

Charophyte meadows have declined and are threatened in shallow coastal habitats, especially in coastal lagoons. Their disappearance has dramatically diminished the ecosystems' diversity, functioning and provisioning services, as they are essential habitats for, e.g., invertebrates (Eveleens Maarse et al. 2024) and fish reproduction (Bučas et al. 2022; Snickars et al. 2010).

Global and national goals require substantial protection and restoration measures of valuable habitats. Charophyte restoration efforts have been made by transplanting charophytes from an existing meadow to a new area (Blindow et al. 2021; Faithfull et al. 2024) or through modifying habitats to create favourable conditions for charophyte species recolonisation (Caffrey et al. 2010; Rip et al. 1992; Rodrigo et al. 2010). However, the methods are expensive to carry out, and success has been uncertain (Faithfull et al. 2024). This study shows that the substrate's physical and chemical properties are specific to *C. tomentosa* , thus potentially limiting the distribution and success of its restoration, particularly in conditions near the limit of its tolerance of salinity, exposure to waves, and competition by other macrophytes. Moreover, the specific microbial communities may play an important role in the nutrient cycling at the sediment–water interface and need further attention to improve our understanding of the role of charophytes in nutrient cycling in eutrophic systems. The results of this study should be utilised in restoring and re‐establishing threatened, declined or vanished *C. tomentosa* habitats to improve ecosystem diversity and resilience (Cardinale 2011) in the Baltic Sea.

## 5 Conclusions

Our results show that *C. tomentosa* prefers fine‐grained substrate. Sediment grain size must be included in planning and implementing restoration and transplantation to achieve good results.

In the sediments from seafloor areas with *C. tomentosa* , the organic content and the total contents of P, N, and C varied less than in the sediments from areas with primarily vascular plants. The lower porewater DIP concentration in the sediments from seafloor areas occupied by *C. tomentosa* raises questions about the importance of *C. tomentosa* habitats in phosphorus cycling. The role of microbial communities in P turnover in these eutrophicated declining habitats calls for further research.

## Author Contributions

**Irma Puttonen:** conceptualization (lead), data curation (lead), formal analysis (lead), investigation (lead), methodology (lead), project administration (supporting), visualization (lead), writing – original draft (lead), writing – review and editing (lead). **Henna Raitanen:** data curation (supporting), investigation (equal), visualization (supporting), writing – review and editing (equal). **Miriam Nystrand:** formal analysis (equal), methodology (equal), validation (equal), writing – review and editing (equal). **Sonja Salovius‐Laurén:** funding acquisition (lead), project administration (lead), resources (lead), visualization (equal), writing – review and editing (equal).

## Funding

This research has received funding from the European Commission LIFE Programme, grant number LIFE20 IPE/FI/000020. Sonja Salovius‐Laurén has received funding from Åbo Akademi University Foundation.

## Conflicts of Interest

The authors declare no conflicts of interest.

## Data Availability

The data used in this study are publicly available in the Zenodo repository (Puttonen et al. 2025), DOI: https://doi.org/10.5281/zenodo.16939316.
